# Gotta Trace ‘em All: A Mini-Review on Tools and Procedures for Segmenting Single Neurons Toward Deciphering the Structural Connectome

**DOI:** 10.3389/fbioe.2019.00202

**Published:** 2019-08-29

**Authors:** Chiara Magliaro, Alejandro L. Callara, Nicola Vanello, Arti Ahluwalia

**Affiliations:** ^1^Research Center “E. Piaggio”, University of Pisa, Pisa, Italy; ^2^Dipartimento di Ingegneria dell'Informazione, University of Pisa, Pisa, Italy

**Keywords:** structural connectome, segmentation algorithm, 3D neuron segmentation, single-cell segmentation, CLARITY

## Abstract

Decoding the morphology and physical connections of all the neurons populating a brain is necessary for predicting and studying the relationships between its form and function, as well as for documenting structural abnormalities in neuropathies. Digitizing a complete and high-fidelity map of the mammalian brain at the micro-scale will allow neuroscientists to understand disease, consciousness, and ultimately what it is that makes us humans. The critical obstacle for reaching this goal is the lack of robust and accurate tools able to deal with 3D datasets representing dense-packed cells in their native arrangement within the brain. This obliges neuroscientist to manually identify the neurons populating an acquired digital image stack, a notably time-consuming procedure prone to human bias. Here we review the automatic and semi-automatic algorithms and software for neuron segmentation available in the literature, as well as the metrics purposely designed for their validation, highlighting their strengths and limitations. In this direction, we also briefly introduce the recent advances in tissue clarification that enable significant improvements in both optical access of neural tissue and image stack quality, and which could enable more efficient segmentation approaches. Finally, we discuss new methods and tools for processing tissues and acquiring images at sub-cellular scales, which will require new robust algorithms for identifying neurons and their sub-structures (e.g., spines, thin neurites). This will lead to a more detailed structural map of the brain, taking twenty-first century cellular neuroscience to the next level, i.e., the Structural Connectome.

**Graphical Abstract:**
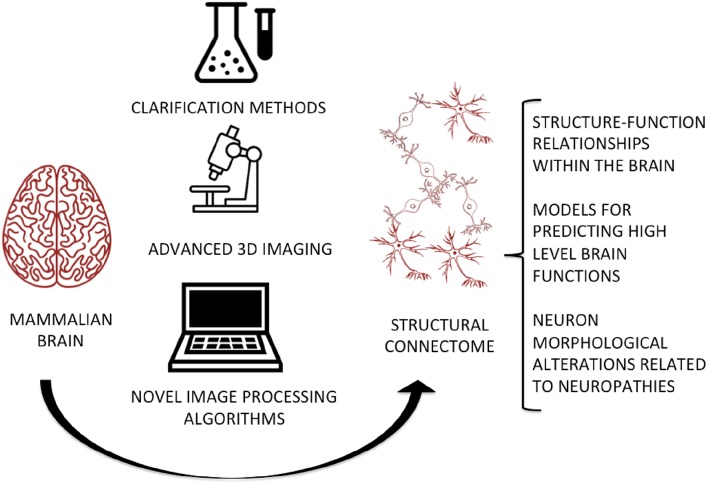
The integration of clarification methods, advanced imaging techniques, and novel image processing algorithms will allow the digitization of a complete and high-fidelity map of the brain at micrometric and even sub-micrometric scales, for predicting and studying the relationships between its micro-circuitry and high-level functions, as well as evaluating abnormal cell morphology in neurodegenerative and neurodevelopmental disorders.

## Brief Historical Perspective

Understanding how the brain works and how it gets sick is one of the biggest scientific challenges of our times (Alivisatos et al., 2012). Deciphering the correspondence between single-neuron morphology and high-level brain function is thought to be the key to unraveling its mystery.

Several projects worldwide are addressing this ambitious goal (Grillner et al., 2016), as summarized in Table 1.

**Table 1:** Principal global initiatives aimed at studying the brain.

	**Starting year**	**Challenge**	**Funding**
European Union Human Brain Project (http://www.humanbrainproject.eu/)	2013	•Simulation and modeling of mice and human brains, based on a detailed neurobiological knowledge of their parts. •Implementation of different infrastructure platforms for high performance computing, medical informatics, neuromorphic engineering and robotics.	300M €
Israel Brain Technologies (http://israelbrain.org/)	2011	•Accelerate brain-related innovation and commercialization.	28M $
Japan Brain/MINDS (http://brainminds.jp/en/)	2014	•Map the brain of a small New World monkey, considered an important step toward gaining better understanding of the human brain.	365M $
US BRAIN Initiative (http://www.braininitiative.nih.gov/)	2013	•Accelerate the development and application of innovative technologies and to construct a dynamic picture of brain function that integrates neuronal and circuit activity over time and space. •Understand how the brain and body create our thoughts, motivations, and feelings.	950M $

Despite the global interest and initiatives, one of the fundamental underlying limitations is still our ignorance about neural architecture and Connectome in the brain. In fact, the complete **Structural Brain Connectome** at the level of synapses has been just reconstructed for the *Caenorhabditis elegans*' nervous system, a worm which has as few as 302 neurons (White et al., 1986). Unfortunately, the complete mapping of more complex brains, such as mammalian ones, is yet beyond our reach. The problem is even more elusive for the human brain, since it comprises an estimated 10^11^ neurons with 10^15^ connections between them. However, it would mark a critical milestone in the worldwide effort to profoundly explore the function of complex neural circuits, as well as to understand fundamental and pathological brain processes.

**Figure 1:**
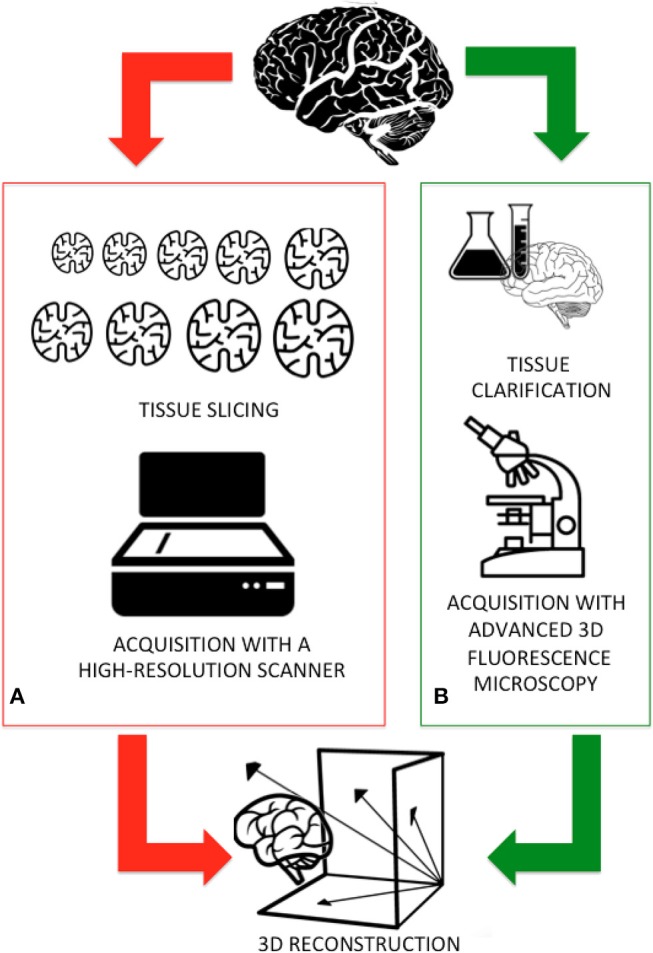
**(A)** The traditional workflow adopted for digitizing an entire brain: the sample is first fixed in formaldehyde and embedded in paraffin and then cut in thin slices. Subsequent slices are collected, acquired using a high-resolution scanner, and finally aligned and reconstructed. **(B)** A new workflow, integrating new methods for processing the samples and advanced 3D imaging will be faster and more accurately deliver the reconstruction of an entire mammal brain.

Light microscopy has long been one of neuroscientists' cardinal tools for studies of cellular morphology and brain cyto-architecture (Wilt et al., 2009). Conventional microscopy is limited by the interaction of light with biological tissues (i.e., scattering and attenuation) and their intrinsic heterogeneity. Indeed, traditionally brain tissue was cut in 10–20 μm-thick slices for ensuring that only a small fraction of photons are scattered (Pawley, 2010). Figure 1A sketches the workflow adopted by the Human Brain Project researchers for digitizing an entire human brain. Firstly, a 65 year old female human brain embedded in paraffin was cut into 7,400 individual slices, each measuring 20 μm-thick. These histological samples were mounted on slides, stained to detect the cell structures, acquired with a high-resolution flatbed scanner, painstakingly aligned, and reconstructed, thus allowing the digitization of an entire human brain down to the cellular level. Although the procedure results in high image quality and diffraction-limited resolution, it is costly, laborious, and involves tissue deformation and loss (Richardson and Lichtman, 2015).

To overcome these setbacks, 3D fluorescence imaging using laser scanning (e.g., confocal and two-photon microscopy) provides the high spatial resolution necessary to resolve individual neurons and neuronal processes at depths of tens to hundreds of micrometers (Ntziachristos, 2010). These **digital imaging** techniques, flanked by the **clarification methods** recently developed for making tissues essentially transparent (Figure 1B), further increase the depth of penetration of light in samples (Richardson and Lichtman, 2015; Magliaro et al., 2016).

Even after the clarification step, 3D digital images representing neurons in their native arrangement within the brain are a challenge to process for detailing single-neuron morphology and topology and cell-to-cell physical connections. In fact, a robust algorithm or tool performing **3D single-neuron segmentation and tracing** from volumetric images of brain tissue is still lacking. From a historical perspective, the majority of the algorithms performing single neuron reconstructions are primarily focused on sparsely labeled data acquired from the nervous systems of insects or worms (Wang et al., 2011; Quan et al., 2016). Thus their application to this new class of images, usually representing dense-packed neurons typical of mammalian brains, is limited (Chothani et al., 2011; Wang et al., 2017; Hernandez et al., 2018). For this reason, manual segmentation is still considered the gold-standard.

In this perspective, the next sections summarize the tools, techniques, and procedures developed in the past few decades for acquiring and then identifying all the neurons and their connections, inspiring new ideas for mapping a complete mammalian brain Structural Connectome.

## Summary of the Established Principles

The role of cell structure in regulating cell behavior and tissue function is well known (Brown et al., 2008). For instance, studies have shown that neurite arborisation patterns established during development are characteristic for particular neuronal subtypes and relate to function. Neurite arbor size and shape influence the integration of synaptic inputs (Gulledge et al., 2005) and these in turn are regulated by both intrinsic developmental programs and external signals (Wong and Ghosh, 2002; Jan and Jan, 2003).

Moreover, the study of cell shape, complexity and size is also vital for studying the normal development of dendritic and axonal arbors and for documenting neuro-pathological changes. In fact, alterations in neurite morphology have been observed in a number of neuro-pathological conditions including mental retardation syndromes (Anderton et al., 1998; Fatemi et al., 2012).

In view of this, new approaches to reconstruct neurons and micro-circuitry from empirical data will aid neuro-anatomical mapping, as well as generating more accurate models that can be used to bridge the gap between single-cell morphology and complex neural structures, paving the way toward making predictions about higher-level brain organization (Budd and Kisvárday, 2012).

## Current State of the Art

### Segmentation Algorithms

While a detailed description of state of art algorithms for single **3D neuron segmentation and tracing** certainly deserves more than a mini-review, here we give an overview aiming at helping neuroscientists to navigate the plethora of published works on neuron segmentation, while underlining some fundamentals aspects when developing new approaches. A summary of the most popular and/or useful available tools for neuron segmentation is reported in Table 2.

**Table 2:** Principal state-of-art tool for segmenting neurons.

	**Availability**	**URL**	**General overview**	**Modality**	**Sparse/dense**	**References**
Neurolucida	Commercial (free-trial available)	•https://www.mbfbioscience.com/free-trials	Suite of tools for visualization, pre-processing, tracing, segmentation, reconstruction, and post-processing analysis.	Automatic/ Semi-Automatic	Sparse/Dense	MicroBrightField, Inc., Williston, VT; Glaser and Glaser, 1990
Vaa3D	Free	•https://github.com/Vaa3D	Suite of tools for visualization, pre-processing, tracing, segmentation, reconstruction, and post-processing analysis.	Automatic/ Semi-Automatic	Sparse/Dense	Peng et al., 2014
Rivulet	Free	•https://github.com/RivuletStudio/(also available as Vaa3D plugin)	Tool for neuron segmentation, tracing, and reconstruction.	Automatic	Sparse	Liu et al., 2016
Neutube	Free	•https://www.neutracing.com/	Tool for neuron tracing, reconstruction, and visualization.	Semi-Automatic/ Manual	Sparse/Dense	Feng et al., 2015
Neuronstudio	Free	•https://icahn.mssm.edu/cnic/tools.html	Tool for neuron tracing and reconstruction.	Semi-Automatic/ Manual	Sparse	Rodriguez et al., 2008
ManSegtool	Free	•https://mansegtool.wordpress.com/downloads/	Tool for neuron segmentation, reconstruction, and visualization.	Manual	Sparse/Dense	Magliaro et al., 2017b
NeuroGPS	Free	•https://sourceforge.net/projects/neurogps-tree/ (also available as Vaa3D plugin)	Tool for post-processing reconstruction of single neurons.	Automatic	Dense	Quan et al., 2016
Tree2Tree	Free	•http://www.ece.virginia.edu/viva/suvadip_docs/Neuron/research_neuron.html	Tool for neuron segmentation and neural branches reconstruction.	Automatic	Sparse	Basu et al., 2010
TREES	Free	•https://www.treestoolbox.org/download.html	Tool for visualization, tracing, segmentation, reconstruction, and post-processing analysis.	Automatic/ Manual	Sparse/Dense	Beining et al., 2017
G-cut	Free	•https://muyezhu@bitbucket.org/muyezhu/gcut/src/master/	Tool for post-processing identification of single neurons (requires already traced structure).	Automatic	Dense	Li et al., 2019

A great number of semi-automatic/automatic 3D tracing algorithms have been proposed to enable large-scale data collection in recent years (Türetken et al., 2011; Ming et al., 2013; Mukherjee et al., 2013; Xiao and Peng, 2013; Yang et al., 2013; Santamaría-Pang et al., 2015; Acciai et al., 2016; Soltanian-Zadeh et al., 2019), and many of these were supported by hackathon events such as the DIADEM [(DIgital reconstructions of Axonal and DEndrite Morphology) challenge in 2009-2010 (Gillette et al., 2011) and the BigNeuron project in 2005 (Peng et al., 2015)], during which different algorithms were compared in terms of reconstruction quality against a manually-traced gold-standard. Most of these are pipelines, combining several steps of image processing, instead of segmentation algorithms *per se*. In this sense, both semi-automatic and automatic 3D approaches are generally integrated into frameworks that combine pre-processing (e.g., denoising), branch tracing, and post-processing methods. Relevant pre-processing steps comprise image denoising as well as deconvolution approaches. Image denoising techniques may assume a specific model of signal and noise according to adopted acquisition methodology, e.g., gaussian or poisson-like, as well as exploit linear or non-filtering, patch-based, and wavelet denoising (Kervrann et al., 2016). Deconvolution techniques aim at restoring images after distortion by microscopes. This operation is complicated by several factors. In fact, while the imaging system point spread function (PSF) is band limited, image noise is not. As a result, image deconvolution is an ill-posed problem and requires the adoption of regularization approaches. Moreover, the PSF may vary across the sample and the shift-invariance approximation that is often assumed might not be valid. Different models for deconvolution adopt a parametric model of noise and/or signal, thus combining denoising and deconvolution steps (Sarder and Nehorai, 2006).

While some segmentation algorithms give more freedom in the pre-processing steps, some others act in a “black-box” fashion, leading to an easier-to-use tool but with less flexibility. Among them, new solutions supported by the application of deep learning have been proposed (Mazzamuto et al., 2018; Soltanian-Zadeh et al., 2019). However, the need of an expert, as well as of training data for fitting the weights of connections within the artificial neural network, can be extremely expensive. Segmentation steps can be followed by tracing algorithms that are developed to identify axons and/or neural branches. For instance, graph theoretic approaches are proposed to connect locally identified trees and generate neuronal global morphology by optimizing a maximum likelihood global tree measure (Basu et al., 2010).

The most popular tool for neuron segmentation from 3D datasets is Neurolucida (MicroBrightField, Inc., Williston, VT; Glaser and Glaser, 1990). However, it is a commercial tool, and we do believe that one of the fundamentals of twenty-first century cellular neuroscience should rely on open-source sharing of tools and algorithms. In this perspective, the main software for sharing new approaches and compare them with existing ones is certainly Vaa3d [i.e., “3D Visualization-Assisted Analysis,” (Peng et al., 2014)]. Originally developed for the visualization of large amounts of data, Vaa3d became a (i) cross-platform (i.e., Windows, Mac, Linux), (ii) modular (i.e., it is composed of different modules for image visualization, segmentation, data analysis, reconstruction comparison), and (iii) open-source suite for image analysis widely used among researchers. Several algorithms are implemented in this suite, allowing for instantaneous “hands-on” access to published algorithms. Furthermore, a user-friendly interface allows also non-expert users to access a great number of implemented algorithms. Nevertheless, sometimes users need their own pipelines for a successful segmentation, making the use of customizable open-source tools preferable (Nunez-Iglesias et al., 2014; Liu et al., 2016).

The segmented structures obtained have to be stored and shared with other researchers labs. Moreover, researchers find themselves struggling to manage their image data (often terabytes) and remote access is always difficult. For these reasons, living on-line archives (e.g., the HBP share platform, the Allen Brain Atlas, OMERO, and DataBrain Linkert et al., 2010; Magliaro et al., 2017a) have been developed. Data already published and organized following template guidelines (e.g., with the proper metadata) can be periodically added to enrich their content. These tools are fundamental for strengthening collaborations between neuroscientists, promoting networking and increasing cooperation with teams with different backgrounds, thus tackling the mapping of the Connectome. Moreover, such databases can be a valid tool for educational and informational purposes.

### Metrics

The ability of an algorithm to isolate single cells is evaluated comparing its outcomes with the ground truth, i.e., manual segmentation. The comparison is performed on some specified metric, which is basically a rule or a set of rules able to quantitatively define the difference between the reconstruction provided by the algorithm and the manual one. In this way, it is possible to define “how far” the automatic reconstruction is from the ground-truth. The ideal metric should be able to tolerate minor differences but also strongly penalize topological (e.g., splits/merges) and morphological (e.g., missing branches, inaccurate dendrite thickness) disagreements.

Several metrics for assessing neuron reconstructions have been proposed and most of them are implemented in both Vaa3d and MATLAB-based tools (Liu et al., 2016). One of the favorites was defined during the DIADEM challenge (Ascoli, 2009). Briefly, this metric compares two digital reconstructions of the same neuron, evaluating the topological similarity between specific points (i.e., the nodes) of the algorithm outcome and of the gold standard. However, the DIADEM metrics, as well as some others defined in the last few years, for example by Peng et al. (Peng et al., 2011; Liu et al., 2016), cannot be exhaustive for neuron segmentation. This is because they only yield geometrical, skeleton-based information about the neuron at selected points, thus neglecting important volumetric information, such as neurite thickness or soma shape.

Attempts at describing new approaches for taking into account the volumetric features of the neurons have been made [e.g., the surface-to-volume ratio of 3D reconstructions obtained both manually and through a tool/algorithm (Liu et al., 2016; Callara et al., 2018)]. In this direction, new approaches including volumetric information should be defined, leading to the next generation of metrics, in which not only discrete “nodes” in the reconstruction, but the whole segmented neuron is used for the comparison, at both volumetric and topological levels. Obviously, this also implies a shift in the way manual segmentation (and not just tracing) should be done (Magliaro et al., 2017b).

## Highlight of Future Directions

The anatomical mapping of the mammalian brain at the micro-scale is a crucial need of the whole neuroscience community. Here, we review the protocols and tools which can be considered as the building blocks for this ambitious aim. The integration of advanced imaging techniques, clearing protocols and new robust image processing algorithms, e.g., the workflow illustrated in the Graphical Abstract, is essential for delivering a high-fidelity map of neuron morphology and topology as well as their physical connections. The main roadblock in the workflow is doubtless isolating single neurons, since a robust algorithm for 3D neuron segmentation is still lacking. In this context, we would like to enhance the importance of isolating and quantifying the shape and size of single neurons rather than just identifying them from the background within a dataset. Indeed, while some methods have been proposed for the segmentation of neurons from clarified tissues (Mazzamuto et al., 2018), they do not as yet yield volumetric or topological information on single neurons.

Thanks to new technologies and protocols available, the digitization of the Structural Connectome can be enriched with sub-cellular details at a level that could not be previously achieved. In particular, the recent development of new fluorescence-based labeling techniques, such as those exploiting membrane probes, allow plasma membrane staining with excellent contrast in single, and double photon imaging. Among them, MemBright fluorescent membrane probes allow neuronal imaging through live, confocal and Stochastic Optical Reconstruction Microscopy (STORM) microscopy (Collot et al., 2019). These approaches are especially powerful when combined with super-resolution imaging, which can improve the spatial resolution of imaging by over an order of magnitude (i.e., down to tens of nanometers). As an example, whole-cell super-resolution imaging (Legant et al., 2016) and super-resolution optical fluctuation imaging (SOFI) (Duwé and Dedecker, 2017) can be employed for producing images of the full 3D cell architecture with a resolution of 50 nm. An algorithm able to deal with such a variety of datasets will speed up neuro-anatomical mapping in mammalian brains even at sub-micrometric resolution, as well as generating models usable for making predictions about higher-level brain organization. Clearly, an integrated multi-disciplinary approach supported by open science principles could accelerate progress in this challenging field.

## Author Contributions

CM and AC wrote the manuscript and constructed the figures. NV and AA conceived and edited the manuscript.

### Conflict of Interest Statement

The authors declare that the research was conducted in the absence of any commercial or financial relationships that could be construed as a potential conflict of interest.

## Key Concepts

### Key Concept 1 | Structural Brain Connectome

The Structural Connectome is a comprehensive map of all the neuronal cells in their three-dimensional (3D) native arrangement and of their physical connections within the brain. Mapping the brain at cellular and sub-cellular resolution is crucial for fully understanding the mechanisms shaping higher level brain functions and for evaluating the morphological abnormalities related to neuropathies.

### Key Concept 2 | Digital Imaging

3D digital imaging is based on sampling successive points in a focal plane to reproduce the spatial distribution of endogenous or exogenous fluorescent probes within a sample. The resulting continuous fluorescence is collected by a detector and converted into a digital image with discrete grey levels through a process known as quantization.

### Key Concept 3 | Tissue Clarification Methods

The mix of components of small size (e.g., proteins, lipids, and pigments) composing a biological tissue has different refractive indices. As a result, their interaction with light leads to scattering and absorption phenomena, limiting imaging at cellular and sub-cellular resolution to depths of a few micrometres. Clearing methods aim at reducing tissue opacity by homogenizing refractive indexes to limit refraction and diffusion phenomena, without damaging the cells.

### Key Concept 4 | 3D Neuron Segmentation and Tracing

3D segmentation is the process of neuron identification in a digital image stack preserving its volumetric information (e.g., neurite thickness and soma volume). 3D tracing just determines the pathway of the neurites, focusing on arborisation topology. The standard formats for storing and sharing tracing outcomes are *.swc and *.asc files, which provide spatial information at some points of interest (e.g., neuron nodes). No similar standards exist for 3D segmentation.
